# Muscle protein synthesis and muscle/metabolic responses to resistance exercise training in South Asian and White European men

**DOI:** 10.1038/s41598-022-06446-7

**Published:** 2022-02-15

**Authors:** Faris F. Aba Alkhayl, Ahmad D. Ismail, Carlos Celis-Morales, John Wilson, Aleksandra Radjenovic, Lynsey Johnston, Paul Welsh, Naveed Sattar, Jason M. R. Gill, Tom Preston, Stuart R. Gray

**Affiliations:** 1 BHF Glasgow Cardiovascular Research Centre, Institute of Cardiovascular and Medical Sciences, College of Medical, Veterinary and Life Sciences, University of Glasgow, Glasgow, G12 8TA UK; 2 Department of Medical Laboratories, College of Applied Medical Sciences, Qassim University, Buraydah, Saudi Arabia; 3 Faculty of Sports Science and Recreation, Universiti Teknologi MARA, Perlis Branch, Arau, Malaysia; 4 School of Life Sciences, University of Glasgow, Glasgow, UK; 5 Scottish Universities Environmental Research Centre, University of Glasgow, Glasgow, UK

**Keywords:** Physiology, Endocrinology

## Abstract

The aims of the current study, therefore, were to compare (1) free-living MPS and (2) muscle and metabolic adaptations to resistance exercise in South Asian and white European adults. Eighteen South Asian and 16 White European men were enrolled in the study. Free-living muscle protein synthesis was measured at baseline. Muscle strength, body composition, resting metabolic rate, VO_2max_ and metabolic responses (insulin sensitivity) to a mixed meal were measured at baseline and following 12 weeks of resistance exercise training. Free-living muscle protein synthesis was not different between South Asians (1.48 ± 0.09%/day) and White Europeans (1.59 ± 0.15%/day) (*p* = 0.522). In response to resistance exercise training there were no differences, between South Asians and White Europeans, muscle mass, lower body strength or insulin sensitivity. However, there were differences between the ethnicities in response to resistance exercise training in body fat, resting carbohydrate and fat metabolism, blood pressure, VO_2max_ and upper body strength with responses less favourable in South Asians. In this exploratory study there were no differences in muscle protein synthesis or anabolic and metabolic responses to resistance exercise, yet there were less favourable responses in several outcomes. These findings require further investigation.

## Introduction

Skeletal muscle has an important role in health^1^ and low muscle strength is associated with an increased risk of a range of poor health outcomes, such as cardiovascular disease (CVD), cancer, respiratory disease, and all-cause mortality^2^. Furthermore, low muscle strength is associated with higher type 2 diabetes incidence^3,4^. On top of this, the higher CVD risk in people with type 2 diabetes is lower in those with high grip strength^5^. Resistance exercise, which is the most effective way to increase muscle strength, reduces several CVD risk factors and improves glycaemic control^6,7^. This provides compelling evidence that the maintenance of muscle strength/mass is important for metabolic health.

It is interesting, therefore, that muscle and its relationship with metabolic health differ between people from different ethnic groups, with muscle mass/function lower, for example, in South Asians relative to white Europeans^8–11^. South Asians also typically develop diabetes 5–10 years earlier than white Europeans^12^; by the age of 70, 30–40% of South Asians have diabetes^12,13^. South Asians transition through the ‘pre-diabetes’ phase more rapidly than Europeans and once they develop diabetes, at a generally lower BMI than white Europeans, disease progression is faster and microvascular complications develop more rapidly^12^. Whilst a range of innate and environmental factors account for the higher diabetes risk in South Asians^12^ it is clear that body composition is very different in south Asians, compared to white Europeans, with higher fat and lower lean/muscle mass^12^*.* This lower muscle mass has been shown to be associated with the higher risk of diabetes and coronary heart disease in south Asians^14^. and in a recent analysis of UK Biobank data we demonstrated that the attributable risk for diabetes associated with low grip strength was higher in south Asians (3.9 and 4.2 cases per 100 men and women) than in white Europeans (2.0 and 0.6 cases)^15^. This highlights that low muscle strength is associated with a disproportionately large number of diabetes cases in South Asians and indicates that strategies to increase muscle mass and function, such as resistance exercise, may be of particular benefit in south Asians. There are, however, very few studies in this area.

In addition, from a mechanistic point of view, it is interesting that South Asians have lower muscle mass and function. Muscle mass is controlled by the balance between muscle protein synthesis (MPS) and muscle protein breakdown (MPB) with the majority of effects in response to anabolic stimuli driven by changes in MPS^16^. There has so far been no comparison of MPS between South Asians and white Europeans in response to anabolic stimuli, although it has been shown that muscle expression of PI3K and PBK Ser473 phosphorylation, which are part of the signalling pathways involved in controlling MPS^17^, are lower in south Asians relative to white Europeans^8^. It may, therefore, be that MPS is lower in south Asians and that this limits the anabolic and metabolic benefits of resistance exercise.

The aims of the current study, therefore, were to compare (1) free-living MPS and (2) muscle and metabolic adaptations to resistance exercise in South Asian and white European adults.

## Methods

### Participants

Eighteen participants of South Asian (India, Pakistani, Bangladeshi or Sri Lanka) descent and 16 participants of white European descent, who were currently residing in the Glasgow area, completed the study, which took place in Glasgow, United Kingdom. Participants were free from injury, metabolic cardiovascular disease, or coagulopathic condition, normotensive (blood pressure < 150/90 mmHg) and did not take part in any moderate to high intensity aerobic exercise or resistance exercise. The sample size calculation was based on detecting a clinically relevant difference in MPS. Even minor changes in MPS of ~ 10–20% are clinically relevant in the long term^18^ and with 15 participants we are able to detect a change in MPS of 0.2%/day with 80% power at *p* < 0.05. We did not carry out a sample size calculation for changes with exercise and so these findings should be considered exploratory. The study was carried out in accordance with the Declaration of Helsinki and the study was approved by the College of Medical, Veterinary and Life Sciences Ethical Committee (No 200150044) with participants providing writing informed consent.

### Study protocol

During the baseline visit, after an overnight fast, resting metabolic rate (RMR), body composition (MRI), knee extensor maximal torque and rate of torque development (RTD) (during a maximal voluntary contraction (MVC)), maximum oxygen uptake (VO_2max_) and a 5 h mixed meal tolerance test was performed. One repetition maximum (1RM) was measured for the following exercises (leg press, bench press, leg extension, shoulder press, leg flexion, seated row, calf raise, latissimus pulldown and biceps curl). Following this visit physical activity (accelerometer) and dietary intake (7-day food diary) were recorded.

On a separate visit a venous blood sample and a spot urine sample were collected, and participants then consumed 100 g of 70% Atom % ^2^H_2_O. For the next 7 days participants were asked to maintain their normal diet and activity habits. Further urine samples were collected at day 2, 5 and 7. On day 7 participants returned to the lab, for the collection of a vastus lateralis muscle biopsy. This concluded the 7-day period for the measurement of free-living habitual fractional synthesis rate (FSR). After this, participants consumed a second dose of 100 g of 70% Atom % ^2^H_2_O.

Participants then began the 12-week resistance training programme with two training sessions per week. Each exercise session involved participants carrying out 1 set to failure at 80% of 1RM of leg press, bench press, leg extension, shoulder press, leg flexion, seated row, calf raise, latissimus pulldown and biceps curl. The 1RM was re-tested at weeks 4 and 8 and the load re-adjusted accordingly. Attendance at sessions was 100%. Three days after the final training session, after an overnight fast, RMR, body composition, knee extensor maximal torque and RTD and VO_2max_, were recorded and a 5 h mixed meal tolerance test performed. The participants were asked to refrain from any other resistance exercise training for the duration of the study and to maintain their usual physical activity and dietary habits.

### Procedures

#### Resting metabolic rate

RMR was determined over a 25-min period with participants resting in supine position under a ventilated hood, using a Quark metabolic cart (COSMED), with the last 15 min of the data collection period used for the estimation of energy expenditure and carbohydrate and fat oxidation.

#### Body composition

Magnetic resonance imaging (3.0 Tesla Siemens) was utilised to quantify body composition using a whole body scan with subsequent data analysis by AMRA^19^. This scan and analysis gave measures of whole-body lean tissue volume, total, visceral and subcutaneous fat volume, liver fat % and selected muscle (calf and thigh) volumes. The calf and thigh muscle volumes were combined to give a lower body muscle volume.

#### Maximal torque and rate of torque development

During a maximal voluntary contraction (MVC) the maximal isometric torque was measured in the right knee extensor muscles at a knee angle of 90° with force record using a load cell. Participants were secured into the chair with the use of belts. Contractions were repeated 3 times with the highest value used for analysis. From the force data the rate of torque development (RTD) was calculated. The torque at times of 0, 50, 100, 200 and 300 ms was determined to allow the calculation of RTD in each time interval by subtracting the torque at time 0 from the torque at each time point and dividing by the time interval.

#### *VO*_*2max*_

Participants performed a 5-min warm up at 30 watts on cycle ergometer followed by an incremental cycle ergometer test, with workload increasing by 30 Watts every minute, until volitional exhaustion. Expired air was collected throughout. VO_2max_ was established as: (1) and RER > 1.1; (2) within 10 bpm of age related HRmax; and (3) an increase in VO_2_ of < 150 ml/min between the final 2 stages.

#### Mixed meal tolerance test

A cannula was inserted into an antecubital or forearm vein and a resting blood sample taken after a 10 min rest period. Subjects then consume a standard test meal (containing 800 kcal, 37% fat, 47% carbohydrate, 17% protein) made up of a bagel, butter, milk and Complan. Further blood samples were collected 30, 45, 60, 90, 120, 180, 240 and 300 min after the consumption of the meal. Samples were analysed for glucose. Triacylglycerol and insulin using an automated analyser (c311, Roche Diagnostics, Burgess Hill, UK) and commercially available ELISA kits (Mercodia), respectively. The MATSUDA index was calculated as a measure of insulin sensitivity^20^.

#### Muscle biopsy

Muscle biopsy samples (50–100 mg) were collected from the vastus lateralis muscle using an automatic biopsy needle (Bard, 16 gauge). Samples were rinsed with sterile saline and dissected free from any fat or connective tissue before being flash frozen in liquid nitrogen. Samples were then stored at − 80 °C before subsequent analysis.

### Analysis

#### Muscle protein synthesis

Procedures were carried out as described previously^21^. Urine samples were vacuum distilled, and the resulting distillate was used. The isotope ratios 2H:1H were analysed on a liquid water analyser (Los Gatos Research, California). Samples were run alongside five in-house lab standards and International standards (SMOW, SLAP and GISP) to correct delta values to ppm. Muscle tissue was homogenized, shaken and centrifuged at 10,000 g at 4 °C for 10 min. The pellet was washed twice with 500 uL of homogenization buffer and centrifuged at 10,000 g, at 4 °C for 10 min. The pellet was treated twice with 500 µL NaOH, centrifuged at 10,000 g, 4 °C for 10 min and the supernatant was retained. 500 µL 1 M PCA were added to the supernatant and samples centrifuged at 1000 g, 4 °C for 20 min. Pellet (myofibrillar fraction) was washed with 2 mL 70% EtOH and centrifuged at 1000 g, 4 °C for 20 min. Pellet was re-suspended in 1 mL HCL, vortex mixed, frozen dry. Myofibrillar protein isolates (approximately 2 mg) were subjected to gas phase acid hydrolysis (6 mol/L HCl; 150 °C for 4 h) and the resulting amino acids were derivatised as ethoxy carbonyl ethyl esters before analysis of deuterium abundance of protein-bound alanine was measured by GC-P-IRMS. Albumin was extracted from the basal plasma sample and albumin-bound alanine was used as proxy for baseline in order to calculate alanine enrichment in myofibrillar protein.

The fractional synthetic rate (FSR; % day^–1^ or % h^‒1^) was calculated as:

FSR = change of myofibrillar protein-bound alanine enrichment with time/ alanine enrichment estimated from average body water enrichment.

### Statistical analysis

Baseline data were compared between South Asians and White Europeans using unpaired t-tests. Normality of data was checked, and the effects of resistance exercise training were compared between the two ethnic groups via linear regression after adjustment for age (and baseline values of the outcome variable. *p* < 0.05 was accepted as statistical significance. All analysis was carried out using STATA (Version Stata/MP 14.2 for Windows). Data are presented as mean ± SD unless sated otherwise.

## Results

White Europeans were younger than South Asians, but there were no differences in height, weight, BMI or physical activity and diet variables (Table 1). Free living habitual FSR (Table 1) did not differ between the groups. There were no differences in resting energy expenditure (*p* = 0.563) or fat (*p* = 0.347) and carbohydrate (*p* = 0.195) oxidation between south Asians and white Europeans at baseline (Table 2). There were no differences between ethnicities in response to resistance exercise training on energy expenditure (*p* = 0.861), but there were differential responses in carbohydrate (*p* = 0.047) and fat oxidation (*p* = 0.041) (Table 2). VO_2max_ was lower at baseline, in South Asians (*p* = 0.033) and there were differences in responses in VO_2max_ (*p* = 0.008) with resistance exercise training, with a lower response in South Asians. There were no differences in systolic (*p* = 0.217) or diastolic (*p* = 0.659) blood pressure at baseline (Table 2). However, whilst the effect of resistance exercise training on diastolic blood pressure was not different (*p* = 0.638) between south Asians and white Europeans, there were differences in responses in systolic blood pressure (*p* = 0.011), with a lower response in south Asians.

**Table 1:** Demographic, MPS, diet and physical activity data at baseline in south Asian and white European volunteers.

	South Asian (n = 18)	White European (n = 16)
Age (years)	24 ± 4	29 ± 7*
Height (cm)	178 ± 9	179 ± 6
Weight (kg)	77.6 ± 13.5	83.2 ± 8.6
BMI (kg/m^2^)	24.5 ± 3.9	25.9 ± 2.7
Muscle Protein Synthesis (%/day)	1.48 ± 0.09	1.59 ± 0.15
Sedentary time (min/day)	419 ± 201	522 ± 256
Light PA (min/day)	64 ± 27	61 ± 24
Moderate PA (min/day)	47 ± 27	42 ± 17
Vigorous PA (min/day)	6.3 ± 7.7	5.0 ± 5.9
Total energy intake (kcal/day)	9317 ± 2048	8874 ± 2219
Protein intake (g/day)	88 ± 16	85 ± 20
Fat intake (g/day)	98 ± 21	94 ± 25
Carbohydrate intake (g/day)	281 ± 93	252 ± 81

Data are presented as mean (SD)* denotes a significant (*p* < 0.05) difference between the groups at baseline (paired t-test).

**Table 2:** Blood pressure, resting metabolic rate, blood and VO_2max_ data at baseline and after exercise training in South Asian and White European volunteers.

	South Asian (n = 18)		White European (n = 16)	
	Baseline	Post-training	Baseline	Post-training
Energy expenditure (kJ/day)	6719 ± 1020	6702 ± 887	6564 ± 493	6953 ± 807
Fat oxidation (g/day)	73.7 ± 44.1	65.1 ± 36.7	88.1 ± 32.3	98.1 ± 44.4**
Carbohydrate oxidation (g/day)	219.6 ± 89.1	237.9 ± 94.3	178.5 ± 68.2	178.3 ± 91.3**
Systolic BP (mmHg)	129 ± 10	126 ± 11	125 ± 7	120 ± 6**
Diastolic BP (mmHg)	75.2 ± 9.1	72.5 ± 6.4	74.0 ± 6.3	71.4 ± 4.8
Fasting glucose (mmol/L)	5.0 ± 0.5	5.1 ± 0.5	5.1 ± 0.5	4.9 ± 0.5
Fasting insulin (µU/ml)	10.0 ± 7.8	10.3 ± 6.6	8.7 ± 4.1	7.5 ± 3.2
Fasting triacylglycerol (mmol/L)	1.4 ± 0.9	1.4 ± 0.9	1.1 ± 0.5	1.2 ± 0.6
VO_2max_ (ml/kg/min)	34 ± 6	33 ± 6	39 ± 8*	41 ± 7**

Data are presented as mean (SD) * denotes a significant (*p* < 0.05) difference between the groups at baseline (paired t-test) and ** denotes a significant (*p* < 0.05) difference between the groups in response to training (ANCOVA).

**Figure 1:**
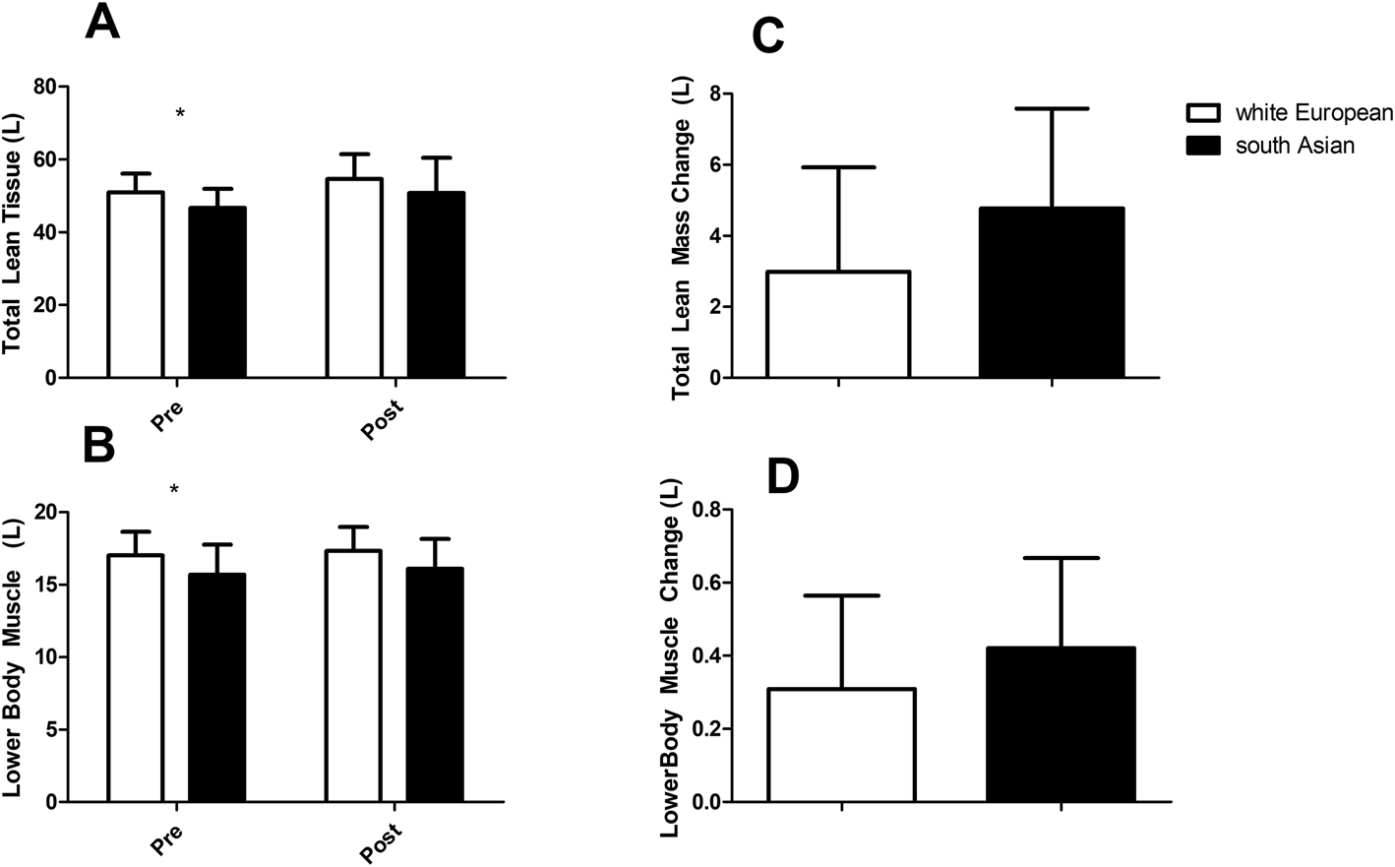
Lean and muscle tissue responses to 12 weeks of resistance exercise training in white Europeans and south Asians. Changes are adjusted for age and baseline values for that variable and are presented as mean (95% CI). * denotes a significant difference between ethnicities at baseline (t-test).

**Figure 2:**
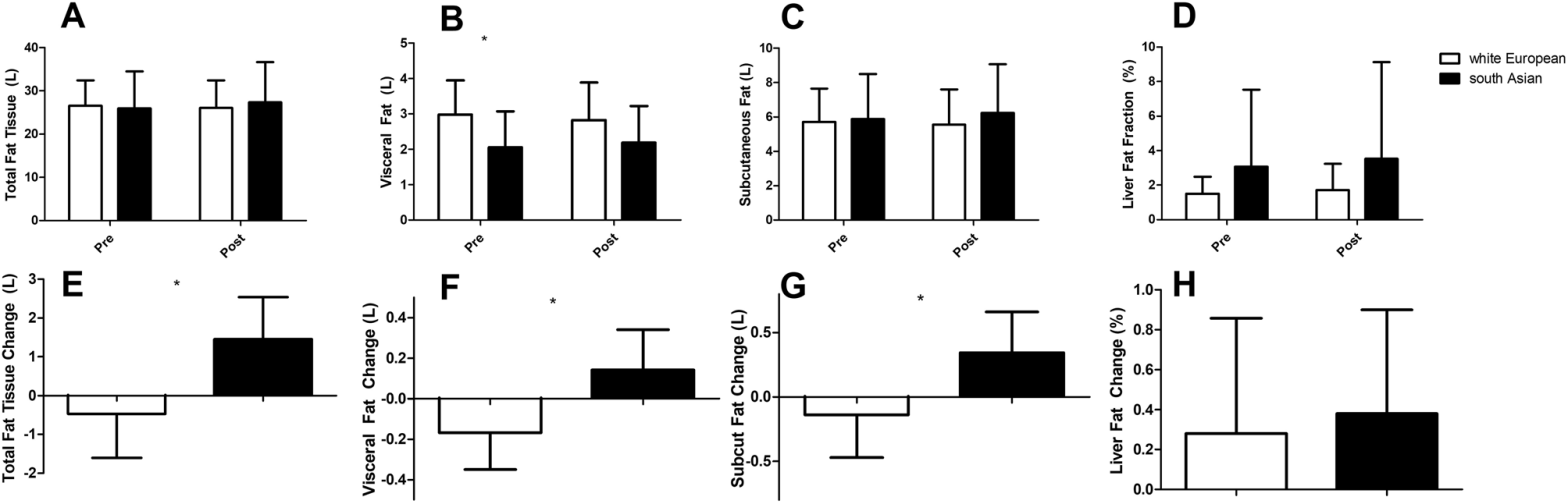
Fat tissue responses to 12 weeks of resistance exercise training in white Europeans and south Asians. Changes are adjusted for age and baseline values for that variable and are presented as mean (95% CI). *Denotes a significant difference between ethnicities in response to resistance exercise training (ANCOVA). Subcut = subcutaneous.

Lower body muscle and total lean tissue data are presented in Fig. 1. Whilst both lower total lean tissue (*p* = 0.031, Fig. 1A) and body muscle (*p* = 0.049, Fig. 1B) were lower in south Asians at baseline there were no differences in response to resistance exercise training (lean mass *p* = 0.415, Fig. 1C, lower body muscle *p* = 0.546, Fig. 1D) between the groups. At baseline there were no differences between groups in total fat (*p* = 0.807, Fig. 2A), subcutaneous fat (*p* = 0.830, Fig. 2C) or liver fat fraction (*p* = 0.213, Fig. 2D), but visceral fat volume (*p* = 0.014, Fig. 2B) was lower in south Asians compared to white Europeans (Fig. 2). Whilst there was no difference (*p* = 0.812, Fig. 2H) in the liver fat fraction in response to resistance exercise training there were differences in responses of total (*p* = 0.008, Fig. 2E), visceral (*p* = 0.029, Fig. 2F) and subcutaneous (*p* = 0.049, Fig. 2G) fat with small declines in white Europeans and increases in south Asians.

**Figure 3:**
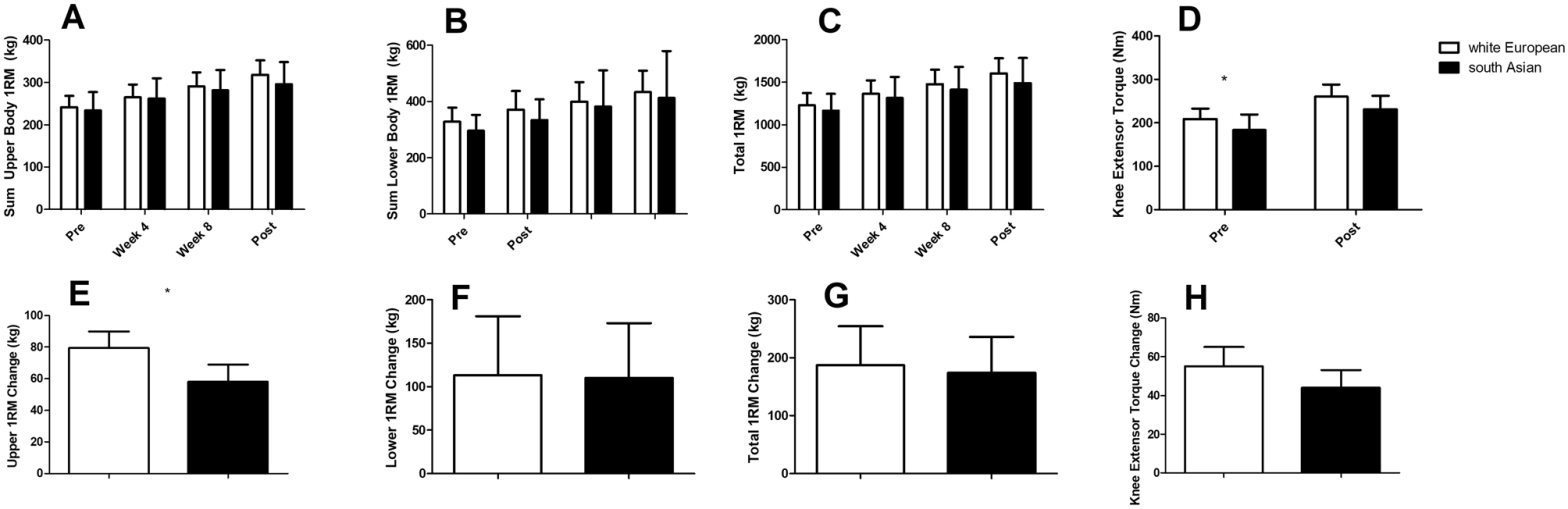
Muscle strength responses to 12 weeks of resistance exercise training in white Europeans and south Asians. Changes are adjusted for age and baseline values for that variable and are presented as mean (95% CI). *Denotes a significant difference between ethnicities in response to resistance exercise training (ANCOVA).

Upper body (*p* = 0.561, Fig. 3A), lower body (*p* = 0.104, Fig. 3B) and total (*p* = 0.177, Fig. 3D) 1RM did not differ between groups at baseline, but knee extensor torque was lower (*p* = 0.025, Fig. 3D) in south Asians compared to white Europeans (Fig. 3). The effect of resistance exercise on upper body 1RM was greater (*p* = 0.015, Fig. 3E) in white Europeans compared to south Asians but there were no differences in the effect of resistance exercise training on lower body 1RM (*p* = 0.947, Fig. 3F), total 1RM (*p* = 0.796, Fig. 3G) or knee extensor torque (*p* = 0.139, Fig. 3H).

**Figure 4:**
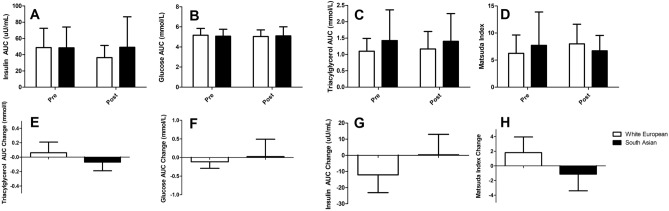
Glucose, triacylglycerol, insulin and Matsuda index responses to 12 weeks of resistance exercise training in white Europeans and south Asians. Changes are adjusted for age and baseline values for that variable and are presented as mean (95% CI).

At baseline there were no differences in fasting glucose (*p* = 0.516), triacylglycerol (*p* = 0.416) or insulin (*p* = 0.438) levels, nor were there any differences in responses to exercise training (glucose *p* = 0.069, triacylglycerol *p* = 0.512 and insulin *p* = 0.07). Insulin (*p* = 0.979, Fig. 4A), glucose (*p* = 0.747, Fig. 4B) and triacylglycerol (*p* = 0.269, Fig. 4C) AUC responses to a mixed meal tolerance test were not different between white Europeans and south Asians at baseline, nor were there differences (insulin *p* = 0.143, Fig. 4E, glucose *p* = 0.642, Fig. 4F and triacylglycerol *p* = 0.696, Fig. 4G) in the responses to resistance exercise training (Fig. 4). There was no difference, between South Asians and White Europeans, in insulin sensitivity, measured via the Matsuda index, at baseline (*p* = 0.461, Fig. 4D) nor in response to exercise training (*p* = 0.051, Fig. 4H) (Fig. 4).

## Discussion

The current study has shown that there are no differences in free-living MPS between young south Asian and White European men. Similarly, were there any differences in the anabolic responses to resistance exercise training with similar increases in muscle and lean mass in South Asians and White Europeans. On the other hand, whilst total and lower body 1RM strength, and knee extensor torque increased to the same extent there was an attenuated increase in upper body 1RM strength in South Asians compared to white Europeans. Similarly, there were less favourable responses in total, visceral and subcutaneous fat, and in systolic blood pressure and VO_2max_ in response to 12 weeks of resistance exercise training. No differences were seen, in response to resistance exercise training, between south Asian and white Europeans in the metabolic responses to a meal ingestion although there was a tendency for a less favourable response in insulin sensitivity, measured via the Matsuda index, in South Asians. One limitation of our study is that we did not have a detailed history of how long participants and/or their families had resided in the UK which means we are unable to explore any potential effects of the time of immigration to the UK on muscle and metabolic health.

There have been very few studies which have previously looked at MPS or the anabolic effects of resistance exercise in south Asians. The current data would indicate in young healthy south Asian men MPS, measured during free-living habitual conditions, is not lower and is, thus unlikely, to underlie the lower muscle mass seen. However, whilst MPS is clearly important for muscle mass, it is not the only factor and does not necessarily associated with longer-term changes in muscle mass^22^. Only one study, to our knowledge has investigated the effects of resistance exercise on muscle mass in South Asians with type 2 diabetes finding no effect of 12 weeks of progressive resistance training on lean body mass or muscle cross-sectional area^23^. This is in contrast to the findings of the present study, where muscle mass appears to increase robustly in South Asians and may reflect differences in the measurement methods employed (DXA/CT vs MRI). It is also worth noting that there were no control groups without exercise training in these studies and so establishing the true effect of exercise is not possible. This is particularly important in a population, people with type 2 diabetes, where muscle mass will be declining^24^.

The study of Misra made no measure of muscle strength, but this has been measured in the work of Knox et al^25,26^. In response to a 6-week progressive resistance training programme, young healthy South Asian men were found to have similar increase in upper body strength, compared to White European men, although increases in lower body strength were attenuated in South Asians. In the current study, the opposite was seen with similar increases in lower body strength, but an attenuated increase in upper body strength. It is hard to make direct comparisons between these studies, as the study of Knox et al. was only 6 weeks in duration, where increases in strength are at an early stage, and used a 3-repetition maximum protocol of only 2 exercises (squat and bench press) to measure strength. Taken together, however, the data does tentatively indicate there may be a slight attenuation in strength gains in south Asian men. It is worth emphasising that this was only one of our strength measures, and the finding may represent a type 1 error. It is also worth noting that robust increases in muscle strength (~ 23% for total 1RM) were still seen in South Asians, thus, resistance exercise should still be recommended to south Asians as the primary method to increase muscle mass and strength.

In both the Knox and Misra studies measure of body fat did not change with resistance exercise training in any groups. The current study found differential changes in total body, visceral and subcutaneous fat between South Asians and white Europeans in response to resistance exercise training, with changes less favourable in South Asians. This is a surprising finding but may be partially supported by the observed difference in the effects of resistance exercise training on carbohydrate and fat oxidation that we observed. The reason for such a differential response to resistance exercise is not clear and warrants further study. Similarly, a differential response in systolic blood pressure was observed, with the decrease smaller in South Asians. It is known that South Asians have impaired endothelial function in forearm resistance vessels and a reduction in bioavailability of nitric oxide at rest and during exercise^27,28^. It may, therefore, be that this observation reflects a reduced response of the vascular system to resistance exercise, but this clearly requires further investigation as the effects of exercise on blood pressure are multifactorial.

As our data demonstrated that increases in muscle mass and strength were similar in south Asians, compared to white Europeans, we may not have expected any differences in metabolic responses to the test meal administered. Indeed, there was no difference in fasting glucose, triacylglycerol and insulin or their responses to the test meal following resistance exercise training. There was, however, a trend for a difference in insulin sensitivity, as measured by the Matsuda index, with a small increase in White Europeans (1.80 95%CI 0.21 to 3.95) which was not seen in South Asians (− 1.13 95%CI − 3.40 to 1.14) which may be worthy of further study. It is worth noting again at this point that the study was not powered to detect differences in metabolic responses to exercise and these findings should be considered exploratory. In the study of Knox and colleagues^25^, where insulin resistance was measured by HOMA-IR, no changes were observed in either White Europeans or South Asians, likely reflecting the short term nature of the study and the measure of insulin resistance employed. On, the other hand the study of Misra et al^23^ found that insulin sensitivity increased and HbA1c decreased, with decreases similar in magnitude to those seen in with European populations in previous meta-analyses e.g.^6^.

In conclusion, the current study has shown that free living MPS is not different between South Asians and White Europeans and that anabolic responses, gains in muscle strength and muscle mass, to resistance exercise broadly similar although these require confirmation in a larger study. We also provide some data which indicates that the cardiometabolic responses to resistance exercise training may be attenuated in South Asians, although again this requires further investigation in a larger study.

## Perspective

Our results indicate that free living muscle protein synthesis is not different between young healthy South Asian and White European men making this an unlikely contributor to the lower muscle mass seen in South Asians. It may be possible that deficits in muscle protein synthesis may exist at different periods of the lifecourse and that there may be differences in muscle protein breakdown. These are worthy of investigation. South Asians are able to mount an anabolic response to resistance exercise training of a similar magnitude as White Europeans with similar increases in muscle mass and strength. However, the beneficial effects of resistance exercise on fitness, blood pressure, body fat and insulin sensitivity appear to be attenuated in South Asians although further work is needed to confirm this.
